# Brain serotonergic fibers suggest anomalous diffusion-based dropout in artificial neural networks

**DOI:** 10.3389/fnins.2022.949934

**Published:** 2022-10-04

**Authors:** Christian Lee, Zheng Zhang, Skirmantas Janušonis

**Affiliations:** ^1^Department of Electrical and Computer Engineering, University of California, Santa Barbara, Santa Barbara, CA, United States; ^2^Department of Psychological and Brain Sciences, University of California, Santa Barbara, Santa Barbara, CA, United States

**Keywords:** artificial neural networks, convolutional neural networks, dropout, regularization, serotonergic, stochastic, anomalous diffusion, fractional Brownian motion

## Abstract

Random dropout has become a standard regularization technique in artificial neural networks (ANNs), but it is currently unknown whether an analogous mechanism exists in biological neural networks (BioNNs). If it does, its structure is likely to be optimized by hundreds of millions of years of evolution, which may suggest novel dropout strategies in large-scale ANNs. We propose that the brain serotonergic fibers (axons) meet some of the expected criteria because of their ubiquitous presence, stochastic structure, and ability to grow throughout the individual’s lifespan. Since the trajectories of serotonergic fibers can be modeled as paths of anomalous diffusion processes, in this proof-of-concept study we investigated a dropout algorithm based on the superdiffusive fractional Brownian motion (FBM). The results demonstrate that serotonergic fibers can potentially implement a dropout-like mechanism in brain tissue, supporting neuroplasticity. They also suggest that mathematical theories of the structure and dynamics of serotonergic fibers can contribute to the design of dropout algorithms in ANNs.

## Introduction

Random dropout is a simple but powerful technique employed in the training of artificial neural networks (ANNs). Its main goal is to improve network regularization and minimize overfitting (Srivastava et al., 2014; Goodfellow et al., 2016). In the standard implementation, the output of a randomly selected set of hidden units is set to zero, and this functional elimination is repeated in each training iteration. The eliminated units neither participate in the current forward pass nor contribute to the backpropagation calculations. As a consequence, the network has a slightly different architecture with each input and cannot heavily rely on any individual neuron (Hinton et al., 2012; Krizhevsky et al., 2012). Conceptually, this technique can be thought of as an efficient approximation of bagging, in which a set of different models is trained on a shared dataset (Goodfellow et al., 2016). In ANNs, the dropout rate is typically 10–50%; the higher rates (40–50%) are common in convolutional neural networks (CNNs) (Geron, 2019).

Artificial neural networks are fundamentally different from biological neural networks (BioNNs), just as the artificial and biological neurons have little in common. In fact, direct mimicking of BioNNs can be counterproductive, as exemplified by the relatively recent transition from the “more natural” sigmoid activation function and to the less biologically realistic but more efficient rectified linear activation function (ReLU) (Krizhevsky et al., 2012). On the other hand, BioNNs are superior to ANNs in their highly sophisticated level of abstraction, which allows them to robustly learn from very small training sets (sometimes a single instance).

The development of ANNs is primarily motivated by practical applications, not by neuroscience. However, each of the two fields has stimulated novel insights in the other. For example, the fundamental architecture of modern CNNs has deep roots in the visual neuroscience of the early 1960s and, in particular, in the notion of a hierarchical system of “receptive fields” (Hubel and Wiesel, 1962; LeMasurier and Van Wart, 2012). On the other hand, recent advances in CNNs have convincingly demonstrated that complex neuroanatomical circuits with many specialized regions (Sporns, 2010) or large-scale oscillations (He et al., 2010) are not necessary for reliable detection and segmentation of objects in complex visual scenes or for human speech recognition (Alzubaidi et al., 2021). Presently, a major effort is underway to make ANNs more “intelligent” (by getting closer to the brain’s ability to operate at a high level of abstraction), which has led to the development of a benchmark dataset, named the Abstraction and Reasoning Corpus (ARC) (Chollet, 2019).

Dropout is peculiar in that it is now a standard and well-validated method in ANN training but it has no obvious counterpart in the biological brain. Logic suggests that BioNNs can also overfit, at the expense of deeper abstractions [as perhaps manifested in savant memory (Bor et al., 2007; Song et al., 2019)]. If a dropout-like mechanism actually exists in neural tissue, it can be expected to be (i) ubiquitously present and have a structure that is both (ii) strongly stochastic, and (iii) unstable at the level of individual neurons, including the adult brain. These requirements may be met by the serotonergic fibers, a unique class of axons described in virtually all studied nervous systems (vertebrate and invertebrate).

In vertebrates, the serotonergic fibers are axons of neurons located in the brainstem raphe complex (Jacobs and Azmitia, 1992; Okaty et al., 2019). These fibers travel in extremely long, meandering trajectories and form dense meshworks in virtually all brain regions (Steinbusch, 1981; Lidov and Molliver, 1982; Foote and Morrison, 1984; Lavoie and Parent, 1991; Vertes, 1991; Voigt and de Lima, 1991; Morin and Meyer-Bernstein, 1999; Vertes et al., 1999; Linley et al., 2013; Migliarini et al., 2013; Donovan et al., 2019). Early estimates have suggested that each cortical neuron in the rat brain is contacted by around 200 serotonergic varicosities (dilated fiber segments) (Jacobs and Azmitia, 1992). The electrophysiological characterization of serotonergic neurons remains grossly incomplete, given their diversity (Okaty et al., 2019). Early studies have reported neurons that fire at remarkably stable rates (Jacobs and Azmitia, 1992), suggesting low information transmission. More recent research has shown that some serotonergic neurons respond to conditions that require learning in uncertainty (Matias et al., 2017), and that serotonin (5-hydroxytryptamine) is fundamentally associated with neural plasticity (Lesch and Waider, 2012). The renewed interest in therapeutic applications of serotonin-associated psychedelics is motivated by the recent findings that these psychedelics can be surprisingly efficient in rapidly boosting cognitive flexibility – thus opening up new opportunities in the treatment of brain disorders associated with cognitive persistence (Vollenweider and Preller, 2020; Daws et al., 2022). Conceptually, serotonin may support “unfreezing” of synapses that may have become “locked in” or “overfitted.”

In addition, recent research has shown that the trajectories of serotonergic fibers are strongly stochastic. Therefore, the number of fiber contacts received by an individual neuron in any brain region is a random event. The mathematical models of serotonergic trajectories are an active area of research (Janušonis and Detering, 2019; Janušonis et al., 2020). Some features of these fibers are captured by the superdiffusive fractional Brownian motion (FBM), an anomalous diffusion process that generalizes normal Brownian motion (Janušonis et al., 2020; Vojta et al., 2020).

Normal Brownian motion describes simple diffusion. Its scientific investigation dates back to the observation of water-suspended pollen grains by Robert Brown (a Scottish botanist) in 1827 and the pioneering theoretical work by Albert Einstein in 1905 (Gardiner, 2010). In normal Brownian motion, the spatial increments in any two non-overlapping time intervals are statistically independent. Diffusing particles that start at the origin produce a gradually widening normal (Gaussian) distribution, the variance of which increases linearly with time. FBM is a major theoretical extension of normal Brownian motion. It can produce three qualitatively different diffusion types, depending on the value of its parameter *H* (the Hurst index, which varies from 0 to 1). The regime at *H* < 0.5 is known as “subdiffusion” (characterized by anti-persistent trajectories, in which two consecutive increments are negatively correlated), the regime at *H* > 0.5 is known as “superdiffusion” (characterized by persistent trajectories, in which two consecutive increments are positively correlated), and normal Brownian motion is recovered as a special case at *H* = 0.5. As *H* approaches one, the trajectories approach straight lines, effectively losing their stochastic character. Diffusing particles described by FBM again follow a normal distribution, but the variance and time are now related by a power-law. Historically, FBM dates back to the data analyses of Harold Edwin Hurst (a British hydrologist) (Hurst, 1951) and the theoretical constructions by Andrey Kolmogorov (Biagini et al., 2010) and Benoit Mandelbrot with John Van Ness (Mandelbrot and Van Ness, 1968).

An important observation for this study is that the paths of individual serotonergic fibers may continuously change, also in the adult brain. Experimental research has demonstrated that serotonergic fibers are nearly unique in their ability to robustly regenerate in the adult mammalian brain after an injury, and that regenerating fibers do not follow their previous paths (Jin et al., 2016; Cooke et al., 2022). Long-term live imaging of serotonergic fibers in intact brains currently poses major technical challenges. However, circumstantial evidence suggests that serotonergic fibers may undergo routine regeneration in the healthy brain. They are extremely long, thin, and not fasciculated, which may result in frequent interruptions because of local tension forces and biological processes, such as microglial activity (Janušonis et al., 2019). This dynamic would continuously generate new fiber paths beyond the interruption points.

**FIGURE 1:**
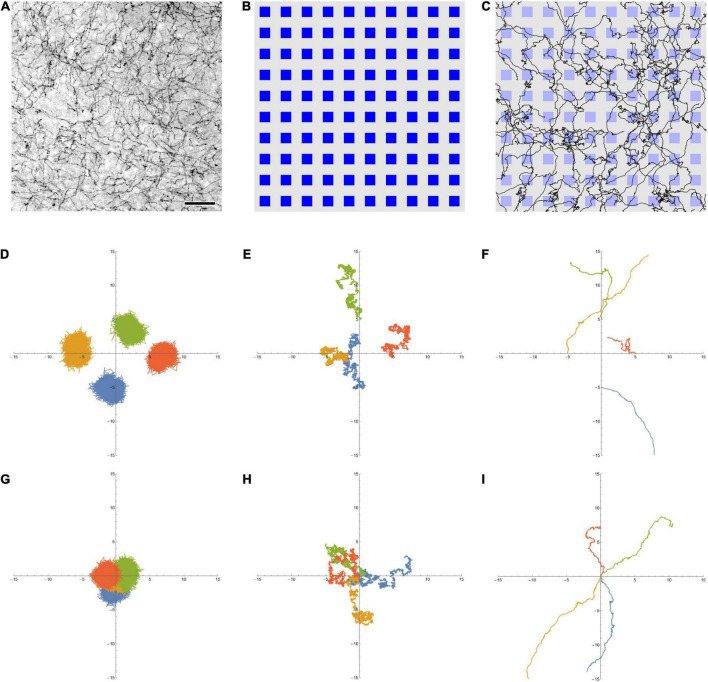
**(A)** Serotonergic fibers in the mouse primary somatosensory cortex, visualized with immunohistochemistry for the serotonin transporter (SERT, 5-HTT). This bright-field image represents three focal levels (in a 40 μm-thick coronal section) that have been digitally merged. The immunohistochemistry was performed as described in Janušonis et al. (2020). Scale bar = 50 μm. **(B)** A 10 × 10 array of artificial neurons, represented by small squares. Their size approximates the size of “typical” brain neurons, on the same physical scale as **(A)** (the side of the squares is around 15 μm). **(C)** A 10 × 10 array of artificial neurons with 50 fractional Brownian motion (FBM) paths (*H* = 0.9, σ = 1, *T* (time length) = 1, Δ*t* (time step) = 0.001) that start at random locations of the array ([0, 1] × [0, 1]). To approximate the microscope field of view, no periodic boundary conditions were used, but the paths were allowed to leave the area and re-enter it (i.e., some paths are represented by more than one visible segment). Note the similarity of the paths to actual serotonergic fibers. **(D,E)** Four FBM sample paths (σ = 1, *T* = 10, Δ*t* = 0.001) starting at points (0, –5), (–5, 0), (0, 5), and (5, 0), with *H* = 0.1 (**D**, subdiffusion), *H* = 0.5 (**E**, normal diffusion), and *H* = 0.9 (**F**, superdiffusion). **(G–I)** Another realization of four FBM sample paths (σ = 1, *T* = 10, Δ*t* = 0.001) starting at the same point (0, 0) to show the relative spread, with *H* = 0.1 (**G**, subdiffusion), *H* = 0.5 (**H**, normal diffusion), and *H* = 0.9 (**I**, superdiffusion). The theoretical standard deviation of the spread is *T^H^* (1.3, 3.2, and 7.9 units in each dimension, for *H* = 0.1, 0.5, and 0.9, respectively).

In summary, serotonergic fibers (Figure 1A) have a number of features that are conducive for a dropout-like mechanism in biological neural tissue. In one scenario, serotonergic fiber contacts may interfere with the normal activity of individual neurons, effectively removing them from the network. Alternatively, these contacts can stabilize the output of individual neurons. *In vitro*, the growth rate of serotonergic axons can be remarkably fast, with long extensions over the course of hours (Azmitia and Whitaker-Azmitia, 1987). To our knowledge, no reliable *in vivo* growth rate estimates are currently available in the healthy adult brain.

In this study, we examined an FBM-based dropout algorithm in simple ANNs, the layers of which (Figure 1B) contained artificial serotonergic fibers modeled as two-dimensional FBM-paths (Figure 1C). We show that the performance of this dropout is comparable to that of the standard dropout. At the same time, the FBM-dropout is considerably more biologically realistic and may stimulate further investigations of its potential in complex, large-scale network architectures.

## Materials and methods

Fractional Brownian motion is a continuous-time and self-similar process, with several stochastic integral representations (Biagini et al., 2010). The distribution of particles that start at the origin is given by a normal distribution, with the mean at the origin and the variance increasing with time as σ^2^*t*^2^*^H^* (where σ > 0 is a constant parameter, *t* is time, and 0 < *H* < 1). At *H* = 0.5, the variance is simply a linear function of time (σ^2^*t*), as expected for normal diffusion. In FBM, the increment correlation between two consecutive time intervals of equal length is given by 2^2*H*−1^−1. Four two-dimensional FBM paths at *H* = 0.1, 0.5, and 0.9, starting at different points to better visualize their individual variability, are shown (Figures 1D–F, respectively). In order to demonstrate the relative spread of paths in the three regimes, four other sample FBM paths, all starting at the origin, are shown for the same *H* values (Figures 1G–I, respectively). In the dropout analyses, FBM paths were generated using the Python *stochastic* (0.6.0) package.

**FIGURE 2:**
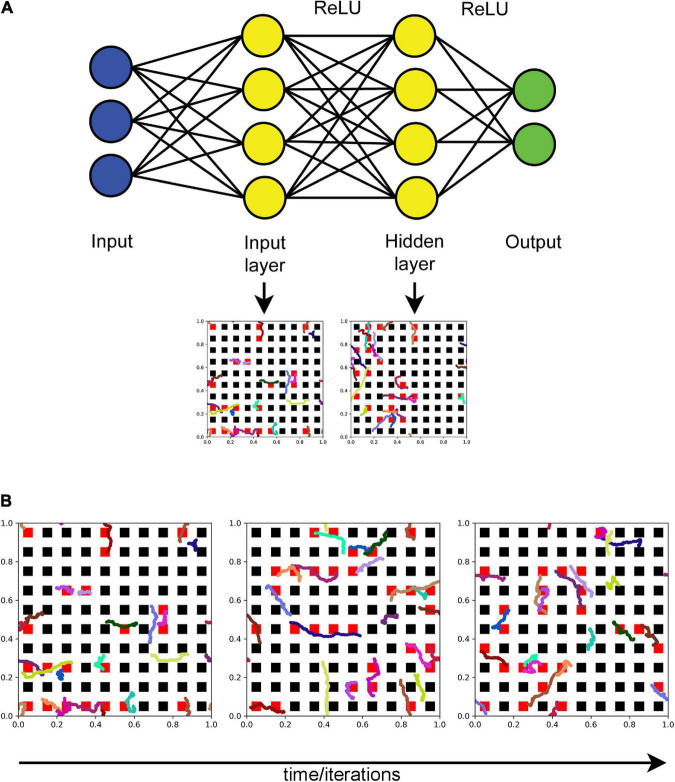
**(A)** The general architecture of the neural network (the actual numbers of neurons are not shown). Fibers move in the input and hidden layers. At each training iteration, all neurons that fall within any of the fiber paths are dropped out. In the standard dropout, each neuron would be dropped out independently of other neurons with a preset probability *p*. **(B)** The dynamics of a set of fibers (randomly color-coded) at 75 **(left)**, 150 **(middle)**, and 225 **(right)** iterations. The neurons that are removed at each step are colored red. All fibers are the same “length” in the sense that they represent equal time intervals (fractional Brownian motion (FBM) paths are fractal and do not have a defined length in the usual sense). *H* = 0.9, *T* = 30, Δ*t* = 1/500, *L* = 50, *s* = 50. Supplementary Video 1 shows the general dropout process.

An FBM-based dropout method (further referred to as the “FBM-dropout”) was tested in a fully connected network consisting of an input layer (with the ReLU activation function), a hidden layer (with the ReLU activation function), and an output layer (Figure 2A). The input and hidden layers were endowed with the Euclidean geometry (i.e., with physical distances), in addition to the standard topological structure (in which only connections and their weights are important). In both layers, neurons were arranged in a square grid (*N* × *N*), extending from 0 to 1 unit in both physical dimensions. In the grid, each neuron was represented by a square with the side of 1/(2*N*) units and adjacent neurons were spaced by the same distance (1/(2*N*) units) in both dimensions (Figure 2B).

The coordinates of two-dimensional FBM paths were modeled as independent one-dimensional paths (with the same *H* and σ = 1). The value of *H* was set at 0.9 based on previous experimental research (Janušonis et al., 2020). For each training epoch, a number (*n*) of long FBM paths were generated in the time interval [0, *T*], and their coordinates at each time-step (Δ*t*) were stored in an array (as the entire trajectories of *n* shorter, *moving* fibers). The variable *n* was used to control the average dropout rate (more fibers result in more neurons dropped out). We note that the dropout rate can also be controlled by adjusting the “length” of the moving fibers or the geometry of the neurons, but these approaches are not equivalent in their statistical structure. Based on an empirical optimization, in the presented analyses we set *T* = *i*_*max*_/10 and Δ*t* = 1/500, where *i*_max_ is the maximal number of training iterations (the actual number of the used iterations may be lower). We note that *T* and Δ*t* refer to the FBM process itself and are not directly related to the network training “time;” the two can be flexibly coupled.

In each iteration (*i* = 0, 1, 2,…), each moving fiber was modeled as a sliding subarray of a long FBM path, representing the time interval [(*i* × *s*) × Δ*t*, (*i* × *s* + *L*) × Δ*t*], where *L* and *s* are positive integers representing the fiber “length” (in the number of points) and the fiber shift in each training iteration (in the number of points), respectively (Figure 2B and Supplementary Video 1). Note that if *s* = *L*, the fiber advances fast and in each iteration starts where it ended in the previous iteration. If *s* < *L*, the fiber “crawls” more slowly, advancing fewer steps and retaining some of its previously occupied positions. In the presented analyses, we set *L* = *s* = 50.

Extremely long FBM paths are computationally expensive and often require supercomputing resources (Janušonis et al., 2020; Vojta et al., 2020), due to their long-range dependence on all previous steps (if *H* ≠ 0.5). To simplify computations, we assumed that at the end of each training epoch the fiber “branches,” initiating a new FBM path at a random point of the last segment, also accompanied by the instant “degeneration” of the previous path. In order to avoid boundary effects (which would require modeling reflected FBM paths (Janušonis et al., 2020; Vojta et al., 2020) but would not be biologically meaningful here), we implemented the periodic boundary conditions (i.e., the fiber never leaves the layer and re-enters on the opposite side when it crosses a border).

The dropout was modeled as follows: if any of the fiber points was located inside a neuron (a geometric square), the output of this neuron was set to zero in this training iteration (Figure 2B and Supplementary Video 1). There was no interaction among the fibers (e.g., the same neuron could be contacted by more than one fiber). The described model is a simplification of biological reality, where serotonergic fibers are attached to a cell body and may instead continuously regenerate with new paths from random interruption points (i.e., they do not actually “crawl” as detached segments). However, the overall dynamic of the model does approximate these biological processes, including axon branching.

All ANN training and testing scripts were written in Python 3 with the PyTorch package (1.11.0). The simulations in the conceptual presentations (Figure 1 and Supplementary Video 1) were written in Wolfram Mathematica 13.0.

## Results

The standard dropout and the FBM-dropout are fundamentally different in that in the former case neurons are turned off independently of other neurons, but in the latter case neurons in close proximity are more likely to be turned off at the same time (because they are more likely to fall on the path of the same fiber). This introduces spatial correlations, which can be tightened or relaxed by controlling the number and “length” of the fibers, their FBM parameter (*H*), their “speed” (*s*), and the geometry of the neurons (e.g., they can be sparsely or densely packed). Despite this more structured dropout, all neurons can get visited at some time during the training (Figure 1 and Supplementary Video 1).

**FIGURE 3:**
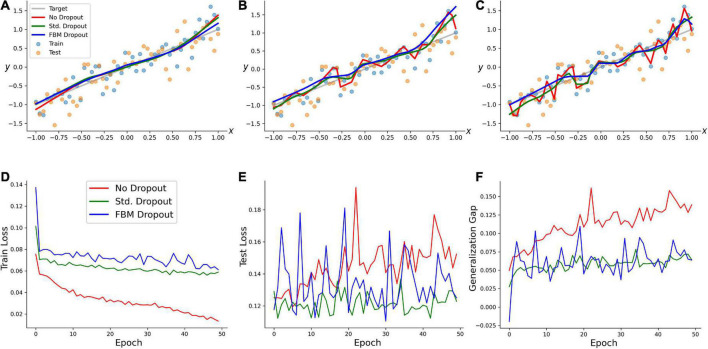
A regression-type model trained with no dropout (red in all panels), with the standard dropout (green in all panels), and with the fractional Brownian motion (FBM)-dropout (blue in all panels). **(A–C)** The fitted curves at 1 **(A)**, 20 **(B)**, and 50 **(C)** epochs. **(D)** The training loss after each training epoch. **(E)** The testing loss after each training epoch. **(F)** The generalization gap (the difference between the testing loss and the training loss) after each training epoch. The network consisted of one input neuron, 100 neurons in the input and hidden layers, and one output neuron. The number of epochs was 50, with 50 mini-batches with one sample each. The Adam optimizer with the learning rate of 0.01 was used. In all conditions, the dropout probability was adjusted to be around 0.2. *N* = 10, *n* = 12, *H* = 0.9, *T* = 5, Δ*t* = 1/500, *L* = 50, *s* = 50. The same dropout parameters were used in the input and hidden layers (the two sets of fibers were independent in the layers).

We examined the performance of the network (with regard to overfitting) in a simple training example. A random set of 50 points in the range of [−1, 1] was generated, and a linearly-dependent second set of points (*y*) was produced, with an additive noise term (*y* = *x* + 0.3ε, where ε has a normal distribution with the mean of 0 and the standard deviation of 1). A network with 100 neurons in the input and hidden layers was trained on this set with no dropout, the standard dropout, and the FBM-dropout. The FBM-dropout strongly outperformed the no-dropout condition and was indistinguishable from the standard dropout (Figure 3). Specifically, the expected regression line was increasingly well approximated by the standard and FBM-dropout models (Figures 3A–C), but the no-dropout model strongly overfitted the training points (the red jagged line in Figure 3C), effectively failing to detect the simple underlying trend (and thus to generalize beyond the training set). This observation was further supported by formal measures that showed that the no-dropout model became increasingly better in capturing the *training* point set (reflected by the low training loss; Figure 3D), but underperformed compared to the other models when presented with a *new (testing)* point set (the high testing loss; Figure 3E). This difference was reflected by the large generalization gap of the no-dropout model (defined as the difference between the models’ performance on the training and new data; Figure 3F).

**FIGURE 4:**
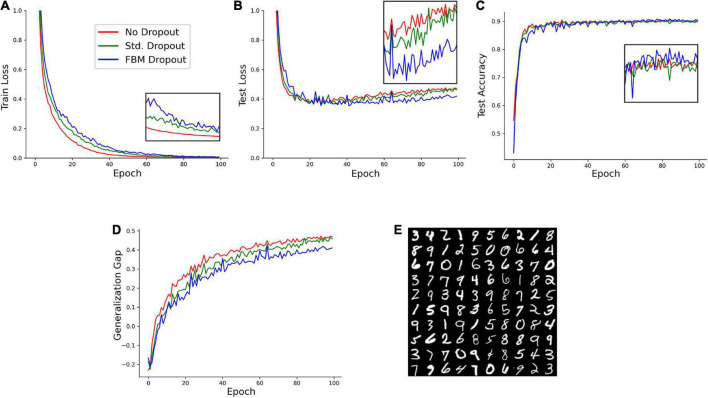
A model trained on an Modified National Institute of Standards and Technology database (MNIST) set with no dropout (red in all panels), with the standard dropout (green in all panels), and with the fractional Brownian motion (FBM)-based dropout (blue in all panels). **(A)** The training loss after each training epoch. **(B)** The testing loss after each training epoch. **(C)** The testing accuracy after each training epoch. The insets show the zoomed-in plot segments after epoch 60. **(D)** The generalization gap (the difference between the testing loss and the training loss) after each training epoch. The network consisted of 784 input neurons (28 × 28 grayscale images of digits), 1,024 neurons in the input and hidden layers, and 10 output neurons. The number of epochs was 100, with 16 mini-batches with 64 samples each. The Adam optimizer with the learning rate of 0.0001 was used. In all conditions, the dropout probability was adjusted to be around 0.2. *N* = 32, *n* = 60, *H* = 0.9, *T* = 2, Δ*t* = 1/500, *L* = 50, *s* = 50. The same dropout parameters were used in the input and hidden layers (the two sets of fibers were independent in the layers). **(E)** Examples of the images used in the training and testing.

We next examined the performance of the network on a reduced set of the Modified National Institute of Standards and Technology database (MNIST) hand-written digits.^1^ In the set, 1,000 training samples and 1,000 testing samples were randomly selected from the larger original set. A network with 1,024 neurons in the input and hidden layers was trained on this set with no dropout, the standard dropout, and the FBM-dropout. The FBM-dropout again performed well compared to the standard dropout (Figure 4). Specifically, the FBM-dropout model slightly lagged behind the other models in capturing the details of the *training* set (reflected by the higher training loss; Figure 4A); however, this resistance to overfitting led to better performance on the *new (testing)* image set, especially after epoch 60 (Figures 4B,C). Consequently, the FBM-dropout model produced a generalization gap that was comparable to, and slightly better than, that of the standard dropout model (Figure 4D).

## Discussion

Dropout was introduced around ten years ago (Hinton et al., 2012; Labach et al., 2019) and since has become a standard technique in the machine learning field. Despite the computational simplicity and effectiveness of random dropout in some ANNs, it has serious limitations in important network architectures. These networks include the powerful CNNs, where random dropout has little effect due to the highly correlated pixels in feature maps (Labach et al., 2019). As ANNs become larger and more complex in their architecture, dropout algorithms are likely to evolve in several directions.

Here, we present an approach that is strongly motivated by neurobiology and is built on recent analyses of serotonergic fibers, led by one of the co-authors (Janušonis and Detering, 2019; Janušonis et al., 2020). We demonstrate the feasibility of this approach in simple, proof-of-concept networks, where it performs at least as well as the standard dropout. However, it has a rich statistical structure which may serve as a toolbox for future improvements in dropout techniques.

Conceptually, the method is simple: the relevant neuron layers are placed in a Euclidean space and enriched with fiber-like entities that move through this space. When a fiber comes into contact with a neuron, the neuron becomes (temporarily) inactive. Computationally, a number of parameters can be easily adjusted, resulting in different dropout statistics. These parameters include the geometry of the layer (the size and shape of the neurons, as well as their spacing which can be deterministic or stochastic) and the fibers themselves, which can differ in their numbers, *H* values, “length,” and “speed.” For example, many short, fast moving fibers with *H* = 0.5 will approximate the standard dropout, but one long, slow moving fiber with *H* > 0.5 will result in strongly correlated dropout events.

An intriguing extension of this method can be produced by adding a third dimension and allowing fibers to move across network layers, as serotonergic fibers do in brain tissue. Tracing studies have shown that a single serotonergic fiber can traverse multiple brain regions, separated by vast anatomical distances (Gagnon and Parent, 2014). This extension is not trivial conceptually, given the topological nature of ANNs, but it may lead to interesting findings. Computationally, it would produce correlated dropout events at different processing levels in the network hierarchy, which might be beneficial in CNNs. It may also find applications in artificial spiking neural networks (SNNs) which already encode spatial and temporal information (Pfeiffer and Pfeil, 2018).

A question arises whether other *H* values in the FBM model or other neurobiologically-inspired dropout models can perform as well. Generally, stochasticity is neither necessary nor sufficient to achieve the regularizing effect in all ANNs (Goodfellow et al., 2016), but the current development of dropout techniques strongly relies on this property (Labach et al., 2019). The *H* values that are close to 0 would not perform well because FBM trajectories would tend to dwell on the same neurons (Figures 1D,G). At the other extreme, the *H* values that are close to one would produce nearly straight trajectories that would cycle through the same subset of neurons (assuming the periodic boundary conditions). As noted, the brain serotonergic system has a number of properties that are particularly well-suited for ANN-like dropout, but it does not rule out a number of other possible mechanisms. In particular, stochasticity has a long history in the analysis in neuron circuits, with a number of recent studies focusing on its fundamental significance and constructive aspects (Geisler and Goldberg, 1966; Srinivasan et al., 1971; Olshausen and Field, 1996; Anton-Sanchez et al., 2014; Shaham et al., 2022; van der Groen et al., 2022).

We note in conclusion that further optimization of dropout techniques may also enrich neuroscience. In particular, the well-described brain regional differences in the density of serotonergic fibers, currently unexplained functionally, might be associated with different levels of plasticity in these brain regions. For example, a high level of plasticity is likely to be beneficial in prefrontal cortical circuits, but such plasticity may be undesirable in brain circuits that control vital organ functions (and may lead to neurological problems). To our knowledge, such analyses have never been carried out. Further insights into dropout algorithms based on FBM, as well as on other anomalous diffusion processes, will strongly motivate this experimental research.

## Data availability statement

The Python and PyTorch scripts used in this study are available at https://github.com/deep-deep-learning/fbm-dropout. Further inquiries can be directed to the corresponding author.

## Ethics statement

The animal study was reviewed and approved by IACUC of the University of California, Santa Barbara.

## Author contributions

SJ proposed that functional similarities may exist between the stochastic organization of brain serotonergic fibers and ANN dropout (as a part of his larger research program), produced simulations in Figure 1 and Supplementary Video 1, and wrote the first draft of the manuscript. CL implemented the FBM-based dropout, performed the computational analyses, and generated all figure panels (with the exception of Figure 1). ZZ supervised the ANN analyses and edited the manuscript. SJ and ZZ are the Principal Investigators of their respective research programs. All authors contributed to the article and approved the submitted version.

## Footnotes

1
http://yann.lecun.com/exdb/mnist

## Supplementary material

Supplementary Video 1 One layer of artificial neurons with one long fractional Brownian motion (FBM) path (*H* = 0.9, σ = 1, *T* = 3, Δ*t* = 0.002) that starts at the center of the array ([0, 1] × [0, 1]) and conforms to the periodic boundary conditions (i.e., the array is topologically a torus: if a fiber leaves the flat array, it re-enters on the opposite side). The neurons that are dropped out are colored red. The yellow flashes represent times when the network receives a training set (e.g., a minibatch), with the following weight update. Generally, the model allows for flexible temporal coupling between the two processes.
